# Characteristics of internal oblique muscle strain in professional baseball players: a case series

**DOI:** 10.1186/s13102-022-00510-5

**Published:** 2022-06-25

**Authors:** Shuro Komatsu, Hironori Kaneko, Masaki Nagashima

**Affiliations:** 1 Institute for Integrated Sports Medicine, Kitasato University Kitasato Institute Hospital, 5-9-1 Shirokane, Minato-ku, Tokyo, 108-8642 Japan; 2 Department of Orthopedic Surgery, International University of Health and Welfare Mita Hospital, 1-4-3 Mita, Minato-ku, Tokyo, 108-8329 Japan; 3 Department of Orthopaedic Surgery, School of Medicine, International University of Health and Welfare, 4-3 Kōzunomori, Narita city, Chiba, 286-8686 Japan

**Keywords:** Professional baseball, Muscle strain, Internal oblique muscle, Trunk side strain

## Abstract

**Background:**

Internal oblique muscle strains can develop in professional baseball players, rendering the players unable to continue playing for a certain period. However, the characteristics of this injury are not well known. The purpose of the present study was to investigate the details of the injury and the post injury course of internal oblique muscle strain in professional baseball players.

**Methods:**

The subjects were members of a single Japanese professional baseball team with a total of 188 players (81 fielders and 107 pitchers) who developed internal oblique muscle strains from January 2012 to December 2021. The diagnosis of muscle strain was made on the basis of local pain and magnetic resonance imaging findings. The incidence of internal oblique muscle strain, the details of the site of the injury, and the time to return to play were examined.

**Results:**

There were 28 cases in 23 players (12.2%) of internal oblique muscle strain. The players were 16 fielders (24.7%) and 7 pitchers (7.5%), with a significantly greater incidence in fielders (*p* = 0.001). Although internal oblique muscle strain was more common on the side contralateral to the batting or pitching side, it occurred on either side. Most of the injury sites were at the region of the muscle insertion to the lower ribs. At a mean time of 36.5 months after the initial injury, 5 players (21.7%) developed another internal oblique muscle strain. The mean time to return to play was 27.7 ± 9.7 days (range, 4–53 days).

**Conclusions:**

Baseball players who have symptoms at the side of the trunk should be regarded as having possible internal oblique muscle strain, and proactive examination should be considered.

## Background

Muscle strain is one of the major sports injuries for professional baseball players [1, 2]. Many professional players are removed from the list for official matches as a result of muscle strains, which can place a significant burden not just on the players themselves, but also on their teams. The majority of muscle strains occurring as a result of sports activities are generally injuries to the leg muscles, such as hamstring injuries [3, 4]. In baseball, the most common cause for athletes to miss Major League Baseball (MLB) games is reported to be hamstring strain, and the second most common is abdominal oblique muscle strain, with the number of cases increasing each year [2, 5]. Although abdominal oblique muscle strain is rare in other sports, it has also been reported to occur in tennis [6] and cricket [7], both of which are sports in which trunk rotation is a major movement, as in baseball [8].

The internal abdominal oblique muscle is reported to be important in the movements that produce ball velocity when pitching and bat swing speed when batting [5]. In cricket, the activity of the internal abdominal oblique muscle was reported to be higher than that of the external abdominal oblique muscle on electromyography during the fast bowling action [9]. In these movements, the main motion is rotation of the trunk. In the bat swing, rotation of the trunk toward the side contralateral to the batting side occurs, and this movement requires a large action of the internal oblique muscle on the side contralateral to the batting side [5, 10]. The trunk also rotates in the same way during pitching or throwing. It seems likely that the injury can be caused as a result of the high load applied to the internal abdominal oblique muscle during this movement.

There are some reports of abdominal oblique muscle strain or side strain in MLB players in the United States [11, 12]. However, these reports included the external abdominal oblique muscle and others, and there are few reports limited to the internal abdominal oblique muscle. Several issues remain unclear, such as the details of the injured site and the side of internal oblique muscle strain, the post injury course, and the difference between fielders and pitchers. The present study investigated the incidence and site of internal oblique muscle strain and the time to return to play in a single professional baseball team.

## Methods

This was a retrospective study. Of 188 players (81 fielders and 107 pitchers) who were members of a single Japanese professional baseball team between January 2012 and December 2021 and included full and partial participations, there were 23 players (16 fielders and 7 pitchers) of internal oblique muscle strain during the 10-year period, thus affecting 12.2% of all players. And 5 of the 23 players (4 fielders and 1 pitcher) suffered a second internal oblique muscle strain within the same period. Those who sustained internal oblique muscle strain were included. There were no players who were affiliated with the team for less than one season. The details of the mechanism of injury, such as hitting and pitching, the injured side, the site of the injury, and the time to return to play, were investigated in these cases. For players who had a second injury, the period between the initial injury and the second injury and the injured side were investigated. All medical records were from the baseball team and there were no uncounted players.

### Diagnosis of internal oblique muscle strain

**Fig. 1:**
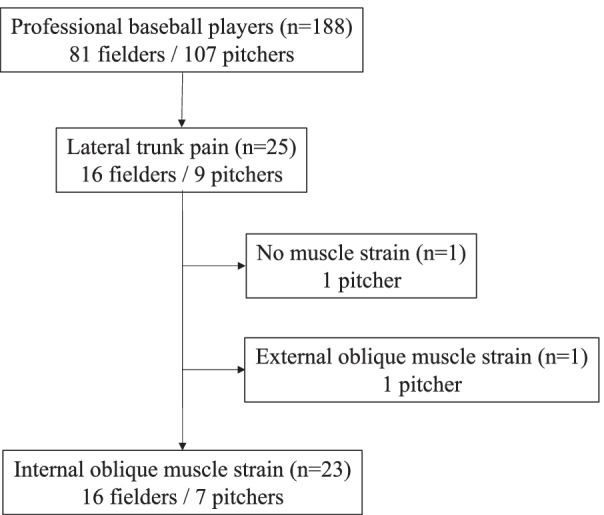
Flowchart of the diagnosis of internal oblique muscle strain

In the present study, a diagnosis of internal oblique muscle strain was made on the basis of medical history and local tenderness, as well as the magnetic resonance imaging (MRI) findings with fat suppression showing high signal intensity in the same area in all cases [12]. MRI was performed when the player could not play for more than one day due to the pain in the lateral trunk. During the observational period, 25 players had the pain in the lateral trunk, so MRI was performed in all 25 players; 1 player had no evidence of muscle strain on MRI, 1 player had external oblique muscle strain, and 23 patients were diagnosed with internal oblique muscle strain (Fig. 1). Although MRI findings were assessed by experienced orthopedic surgeons and radiologists, the reading results shown in the present study were basically those of the radiologist.

### Classification of the muscle injury site on MRI

**Fig. 2:**
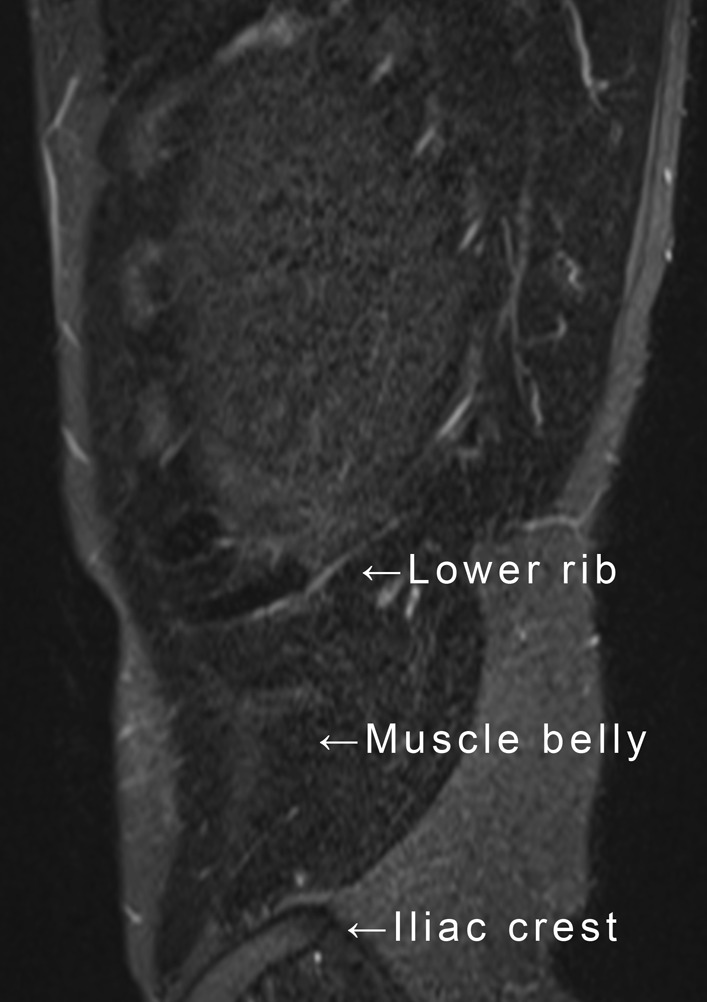
Classification of the internal abdominal oblique muscle injury site

The site of the muscle injury was assessed using sagittal and coronal MRI. MRI of the internal oblique muscle was conducted using a 3.0-T scanner (MAGNETOM Lumina; Siemens Health Care, Erlangen, Germany). Setting for sagittal Short Tau Inversion Recovery (STIR) imaging were: repetition time, 1040 ms; echo time, 80 ms; echo train length, 256; slice thickness, 6.0 mm; field of view, 420 × 420 mm; matrix size, 400 × 400; and number of excitations, 1. Setting for axial STIR imaging were: repetition time, 676 ms; echo time, 72 ms; echo train length, 192; slice thickness, 3.0 mm; field of view, 420 × 420 mm; matrix size, 320 × 320; and number of excitations, 1. The internal oblique muscle originates from the iliac crest and its surroundings (inguinal ligament and thoracolumbar fascia) and inserts into the lower ribs and their surroundings (rectus abdominis sheath and levator testis muscle). The site of muscle injury was classified into the lower rib area, the muscle belly, and the iliac crest area for assessment (Fig. 2).

On sagittal MRI with fat suppression, the site of muscle injury was classified as the lower rib area, the muscle belly, and the iliac crest area.

### Injured side

The injured side was assessed in the case of injuries caused by batting, throwing, or pitching. In the present study, it was more straightforward to use the dominant batting side or the dominant arm as a reference point. In fielders, a contralateral injury was defined as being opposite the dominant batting side. As for pitchers and fielders throwing, a contralateral injury was defined as being opposite the dominant arm [5].

### Return to play

For the present study, return to play was defined as participation in an official game. The time to return to play was defined as the period from injury to return to official games, even in the case of players who were able to play for a while after their injury. If the season ended before a player returned to official games, the time to return to play could not be measured, and the player was therefore excluded from this evaluation.

### Statistical analysis

The frequency of internal oblique muscle strain and the time to return to play were compared between fielders and pitchers**.** using the χ^2^ test and Student’s *t*-test, respectively. The level of significance was set at *p* < 0.05. All statistical analyses were performed with BellCurve for Excel (Social Survey Research Information Co., Ltd., Tokyo, Japan).

This study was approved by our institutional review board (file no. 17030).

## Results

Details of the 23 players are shown in Table 1. The mean age of injured athletes was 28.3 years, and their mean body mass index was 26.7 kg/m^2^. Including the initial and second injuries, there were 28 cases in 23 players (20 cases in 16 fielders and 8 cases in 7 pitchers) of internal oblique muscle strain during the 10-year period. The rate of occurrence of internal oblique muscle strain was 24.7% in fielders and 7.5% in pitchers, with the incidence significantly higher in fielders (*p* = 0.001).

**Table 1:** patient demographic and clinical data

Case	Age (year)	Position	Injured month	The interval between the injury onset and MRI scan (day)	Mechanism of injury	Dominant side	Injured side	Injury site on MRI	Return to play (day)	Second injury
1	28	Fielder	Feb	3	Batting (game)	Left	Contralateral	Lower rib area	13	–
2	25	Pitcher	Feb	21	Pitching (practice)	Right	Contralateral	Lower rib area	53	–
3	22	Pitcher	Mar	1	Pitching (practice)	Right	Contralateral	Lower rib area	Season interruption	–
4	31	Fielder	Mar	2	Batting (practice)	right	Contralateral	Lower rib area	20	–
5	34	Fielder	Mar	1	Batting (practice)	Left	Dominant	Lower rib area	34	–
6	30	Fielder	Apr	1	Batting (game)	Left	Contralateral	Lower rib area	34	–
7	31	Fielder	Apr	1	Batting (game)	Left	Contralateral	Lower rib area	21	–
8	30	Pitcher	Apr	11	Pitching (game)	Right	Dominant	Lower rib area	35	+
9	30	Pitcher	May	5	Unclear	Right	Dominant	Lower rib area	28	–
10	22	Fielder	May	0	Batting (practice)	Left	Contralateral	Lower rib area	23	–
11	27	Pitcher	May	1	Pitching (game)	Right	Contralateral	Lower rib area	28	–
12	33	Fielder	May	0	Batting (game)	Left	Contralateral	Lower rib area	36	–
13	31	Fielder	May	4	Batting (game)	Right	Contralateral	Lower rib area	29	–
14	27	Fielder	Jun	0	Batting (practice)	Right	Contralateral	Lower rib area	8	+
15	40	Pitcher	Jul	1	Pitching (game)	Left	Contralateral	Lower rib area	25	–
16	32	Fielder	Jul	0	Batting (game)	Left	Dominant	Lower rib area	28	–
17	23	Fielder	Aug	4	Batting (game)	Right	Contralateral	Lower rib area	31	+
18	29	Fielder	Aug	1	Batting (game)	Right	Dominant	Lower rib area	28	+
19	27	Fielder	Sep	1	Batting (game)	Right	Dominant	Lower rib area	24	+
20	23	Fielder	Sep	2	Throwing (practice)	Right	Contralateral	Lower rib area	Season ended	–
21	25	Fielder	Oct	1	Batting (game)	Left	Contralateral	Lower rib area	Season ended	–
22	31	Pitcher	Oct	2	Pitching (game)	Left	Contralateral	Lower rib area	Season ended	–
23	21	Fielder	Nov	1	Batting (game)	Right	Dominant	Lower rib area	Season ended	–

**Fig. 3:**
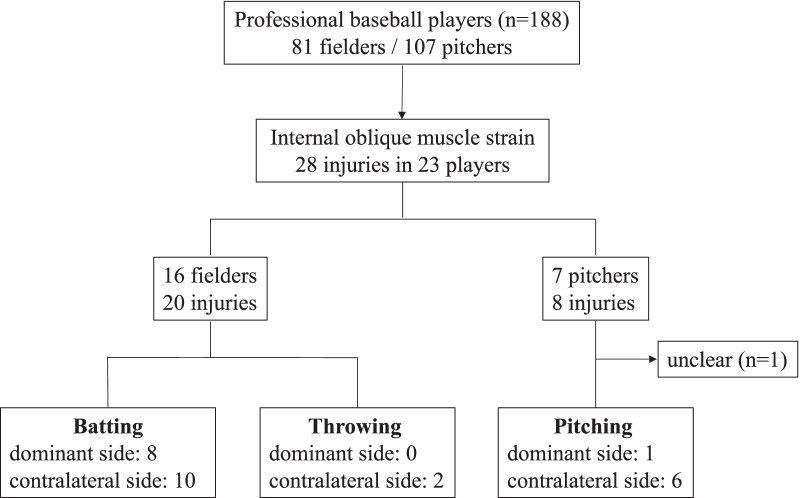
Flowchart of the injury mechanism and the injured side

**Fig. 4:**
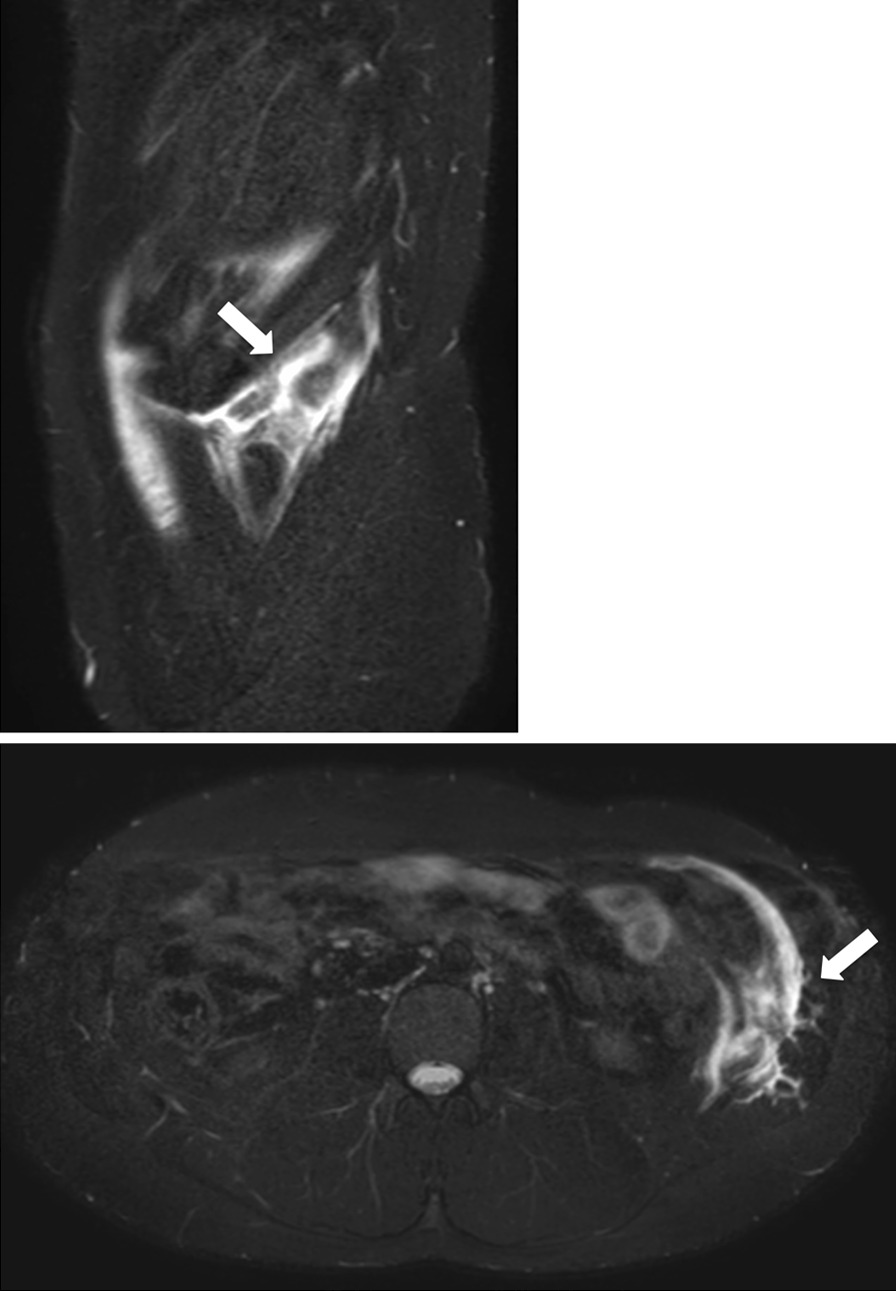
Sagittal and Axial MRI of internal oblique muscle strain. On sagittal and axial MRI with fat suppression, an area of high signal intensity is visible in the lower rib area (white arrow)

As for the mechanism of injury, including initial and second injuries, 18 injuries in 14 fielders occurred during batting, and 2 injuries occurred in 2 fielders during throwing. As to the injured side, 10 of the 18 injuries occurred during batting on the contralateral side and 2 occurred during throwing, contralateral to the throwing side. With pitchers, all but 1 case occurred during pitching, and 6 injuries occurred contralateral to the pitching side (Fig. 3). In one pitcher, the mechanism of injury was unclear, and the injury occurred on the dominant side. In the injury site classification, all but 2 injuries were located in the lower rib area (Fig. 4), and the other 2 injuries that were the second injury were located in the muscle belly. All cases were treated conservatively and returned to play. None of the cases have received corticosteroid or platelet-rich plasma injections.

Eight of 28 cases were excluded from the measurement of time to return to play because the season ended before they returned to official games. The overall mean time from the injury to return to play in a game was 27.7 ± 9.7 days (range, 4–53 days), and the mean was 23.8 ± 9.5 days (range, 4–36 days) in fielders and 33.8 ± 11.3 days (range, 25–53 days) in pitchers, with no significant difference between them (*p* = 0.06).

### Second injury

The mean time from the initial injury to the final evaluation for 23 players was 36.5 months (range, 3–89 months). There were 5 players (4 fielders and 1 pitcher) with a second internal oblique muscle strain during the period. The second injury rate was 21.7%. The mean time between the initial and second injuries was 20.2 months. Four of the 5 cases injured the side opposite to the initial injury. All cases were treated conservatively again and returned to play.

## Discussion

In the present study, 12.2% of players developed internal oblique muscle strain in a single Japanese professional baseball team, with the incidence significantly higher in fielders. Most injury sites were at the region of the muscle insertion to the lower ribs. A mean of 36.5 months after the initial injury, 21.7% of the players were injured again, which is by no means a small proportion. A mean time of 27.7 days was needed to return to play.

In the MLB, 393 cases of abdominal muscle strain were reported to occur over a period of 20 years, with the number increasing every year [5]. In addition, the re-injury rate was reported to be 12% [5], which is lower than in the current study. The incidence of internal oblique muscle strain in fielders and pitchers was reported to be almost equal in the MLB, but in the present study, the incidence was significantly higher in fielders. In the present study, MRI was indicated for players who had difficulty continuing to play. It was possible that the different methods for diagnosing internal oblique muscle strain affected these results.

In the present study, most injury sites were at the region of the muscle insertion to the lower ribs. As noted by Connel et al., this was because the region is weak and vulnerable to injury [12]. They also reported that most of the pain in the lateral trunk of athletes might be internal oblique muscle strain [12]. Athletes who complain of pain in this region should be considered for proactive examination for suspected internal oblique muscle strain.

Regarding the injured side, it has been reported that 78% of pitchers and 70% of fielders developed abdominal oblique muscle strain on the contralateral batting and pitching sides in MLB [5]. In Australian and English first-class cricket fast bowlers, the injured side of side strain of all 108 bowlers was reported to be the contralateral side [13]. Furthermore, in Australian first-class cricket fast bowlers, all 10 internal oblique muscle strains diagnosed by MRI were on the contralateral side [7]. A previous study that used electromyography to examine the activity of abdominal muscles during baseball pitching reported that the muscles on the contralateral side were more active than those on the dominant side [14]. In the present study, although internal oblique muscle strain was more common on the side contralateral to the batting side or the pitching side, it occurred on either side, especially in second injury cases, most of which were on the side opposite to the initial injury. The details of the mechanism of abdominal oblique muscle strain are still not fully known, and further studies are needed in this area.

It has been reported that the time to return to play was approximately 4–5 weeks for athletes after a side strain [15]. In the present study, fielders had a shorter time to return to play than pitchers, although the difference was not significant. Similarly, a prior study reported earlier return to play in fielders than pitchers [5]. Fielders may return to play earlier because they can participate at least partially in a game through pinch batting, pinch running, or defense only, even if they have not returned to their full performance level.

The present study has some limitations. First, it included a small number of cases limited to a single Japanese professional baseball team. Since only one team was surveyed in this study, the results may have been influenced by the circumstances and policies of this team. Second, all cases of internal oblique muscle strain could not be examined. Only the injuries that occurred during the season were examined. And, it is possible that we may have missed a minor internal abdominal muscle strain which did not make playing impossible for more than 1 day. Third, we were not able to assess the severity of the internal oblique muscle strain in this study. Therefore, there is a large variation in the time from injury to return to play. Fourth, as an indicator of return to play, the date of available to play should be evaluated, but the record was not available and the date of return to play in an official game were evaluated in this study. The date of return to play in a game could have been influenced by the importance of the player on the team. We intend to investigate the occurrence and recurrence of internal oblique muscle strain in order to gain a fuller understanding of this injury and to investigate preventive measures.

## Conclusions

In a single Japanese professional baseball team, there were 28 cases of internal oblique muscle strain in 23 players (12.2% of all players) during a 10-year period. Baseball players who have symptoms at the side of the trunk should be regarded as having possible internal oblique muscle strain, and proactive examination should be considered.

## Data Availability

The datasets used and/or analysed during the current study are available from the corresponding author on reasonable request.

## Abbreviations

MLB: Major League Baseball
MRI: Magnetic resonance imaging
STIR: Short tau inversion recovery
